# Ultrathin PEDOT:PSS Enables Colorful and Efficient Perovskite Light‐Emitting Diodes

**DOI:** 10.1002/advs.202000689

**Published:** 2020-04-13

**Authors:** Jianxun Lu, Wenjing Feng, Guanding Mei, Jiayun Sun, Chuanzhong Yan, Di Zhang, Kebin Lin, Dan Wu, Kai Wang, Zhanhua Wei

**Affiliations:** ^1^ Institute of Luminescent Materials and Information DisplaysCollege of Materials Science and Engineering Huaqiao University Xiamen 361021 P. R. China; ^2^ Academy for Advanced Interdisciplinary Studies Southern University of Science and Technology Shenzhen 518055 P. R. China; ^3^ Department of Electrical and Electronic Engineering Southern University of Science and Technology Shenzhen 518055 P. R. China

**Keywords:** light‐emitting diodes, outcoupling efficiency, perovskites light‐emitting diodes, poly(3,4‐ethylenedioxythiophene):poly(styrene sulfonate)

## Abstract

Recently, metal halide perovskite light‐emitting diodes (Pero‐LEDs) have achieved significant improvement in device performance, especially for external quantum efficiency (EQE). And EQE is mostly determined by internal quantum efficiency of the emitting material, charge injection balancing factor (η_c_), and light extraction efficiency (LEE) of the device. Herein, an ultrathin poly(3,4‐ethylenedioxythiophene):poly(styrene sulfonate) (UT‐PEDOT:PSS) hole transporter layer is prepared by a water stripping method, and the UT‐PEDOT:PSS can enhance η_c_ and LEE simultaneously in Pero‐LEDs, mostly due to the improved carrier mobility, more matched energy level alignment, and reduced photon loss. More importantly, the performance enhancement from UT‐PEDOT:PSS is quite universal and applicable in different kinds of Pero‐LEDs. As a result, the EQEs of Pero‐LEDs based on 3D, quasi‐3D, and quasi‐2D perovskites obtain enhancements of 42%, 87%, and 111%, and the corresponding maximum EQE reaches 17.6%, 15.0%, and 6.8%, respectively.

## Graphical abstract

The popular poly(3,4‐ethylenedioxythiophene):poly(styrene sulfonate) (PEDOT:PSS) hole transporter layer in perovskite light‐emitting diodes will cause some loss of photons and result in limited device performance. Herein, to overcome this problem, an ultrathin PEDOT:PSS is prepared, and performance is successfully improved in 3D, quasi‐3D, and quasi‐2D perovskites.

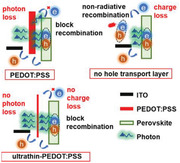

Metal halide perovskite light‐emitting diodes (Pero‐LEDs) have attracted a great deal of interest in recent years due to their huge potential and promising applications in lighting and future‐generation displays.^[^ ^1^, ^2^, ^3^, ^4^, ^5^ ^]^ Their superior optoelectronic properties include high photoluminescence quantum yield (PLQY),^[^ ^6^ ^]^ narrow emission bandwidth, broadly tunable band gaps, low raw materials cost, and simple solution‐based fabrication process.^[^ ^7^, ^8^, ^9^, ^10^ ^]^ Since the first report of room temperature operable Pero‐LEDs in 2014,^[^ ^3^ ^]^ this field has undergone an amazing and rapid growth of device performance. External quantum efficiency (EQE) is one of the most common metrics to evaluate the device performance of Pero‐LEDs, where EQE is the ratio of the number of photons emitted from the device to the number of electrons injected into the device.^[^ ^1^ ^]^ After years of development, EQEs of green,^[^ ^11^ ^]^ visible red,^[^ ^12^ ^]^ and near infrared^[^ ^13^, ^14^ ^]^ emitting Pero‐LEDs have all exceeded 20%, showing great potential in practical application like lighting and displays. However, as for device performance and operational stability, there is still a huge gap between Pero‐LEDs and other mature technologies, like organic LEDs (OLEDs) and quantum dot LEDs.^[^ ^15^ ^]^

To further promote device performance and operational stability of Pero‐LEDs, it is essential to understand how charges are injected and transported in the device, the recombination process is manipulated, and the proportion of radiative recombination to non‐radiative recombination is increased.^[^ ^16^ ^]^ In a typical cell structure of Pero‐LEDs, the perovskite emitting layer (EML) is sandwiched between electron transport layer (ETL) and hole transport layer (HTL) materials. The poly(3,4‐ethylenedioxythiophene):poly(styrene sulfonate) (PEDOT:PSS) is the most commonly used HTL mostly due to its easy solution‐based fabrication process and high charge carrier mobility (0.0128 cm^2^ V^−1^ S^−1^).^[^ ^17^ ^]^ However, the energy level mismatch between PEDOT:PSS (−5.0 to −5.1 eV, the highest occupied molecular orbital (HOMO)) and perovskite (−5.6 to −5.9 eV, valance band maximum (VB_max_))^[^ ^11^, ^18^ ^]^ leads to an inferior hole injection, compared to the electron injection which happens in the interface of perovskite and ETL. For example, the lowest unoccupied molecular orbital (LUMO) energy level of 4,6‐bis(3,5‐di‐3‐pyridinylphenyl)‐2‐methylpyrimidine (B3PYMPM, −3.2 eV) is closer to the conduction band minimum (CB_min_) energy level of perovskite (−3.4 to −3.6 eV).^[^ ^11^, ^18^ ^]^ This imbalance injection of electrons and holes results in a limitation of device performance. There are already some strategies such as adding additives^[^ ^19^, ^20^, ^21^, ^22^, ^23^ ^]^ and solvent treatment^[^ ^24^, ^25^, ^26^, ^27^, ^28^ ^]^ to improve the hole injection process of the interface of PEDOT:PSS and perovskite. Lee et al.^[^ ^21^ ^]^ applied methylammonium bromide /dimethyl sulfoxide (DMSO) or methylammonium iodide (MAI)/DMSO as co‐additives to increase the hole mobility and reduce the HOMO energy level of PEDOT:PSS, and they finally obtained a maximum EQE up to 10.93%. Song et al.^[^ ^23^ ^]^ and Chen et al.^[^ ^22^ ^]^ demonstrated similar methods by introducing MoO_3_ and MoO_3_‐ammonia, respectively into PEDOT:PSS to enhance the hole injection process, and the optimized Pero‐LEDs with MoO_3_ modification showed enhanced EQE and luminance compared to the Pero‐LEDs using a pristine PEDOT:PSS. Yu et al.^[^ ^27^, ^28^ ^]^ treated PEDOT:PSS film with high‐polarity alcohol solvent such as methanol, ethanol, and isopropanol to increase the conductivity, and finally obtained a higher luminance. Besides the imbalanced charge injection process, it needs to be pointed out that the EQE of Pero‐LEDs is also significantly influenced by the thickness of PEDOT:PSS. The relative contribution of outcoupled photons among various power dissipation channels will be tuned as the thickness variation of the PEDOT:PSS. This is partially due to the nonzero of the imaginary part of refractive index at emission spectrum of perovskite, which will induce relatively low light extraction efficiency (LEE). Moreover, the equivalent radiative recombination center location within the perovskite layer will also be influenced as the injection properties change due to the thickness of PEDOT:PSS variation, which play a vital role in the LEE. Unlike the internal quantum efficiency (IQE) which is majorly determined by the material itself, the LEE of Pero‐LEDs is more complicated and influenced by both the material properties and the device structure. All in all, it is highly desirable to develop a simple but effective method to manipulate the PEDOT:PSS layer, increase its hole‐transporting capability, tune its energy level, and improve the overall LEE of Pero‐LEDs.

Herein, we prepared an ultrathin PEDOT:PSS (UT‐PEDOT:PSS) HTL by water stripping method and used it to replace with the conventional thick PEDOT:PSS HTL in Pero‐LEDs. After reducing the film thickness, the ultrathin PEDOT:PSS posed a higher conductivity and a deeper HOMO energy level, and the LEE of Pero‐LED was greatly enhanced. Moreover, we found that the UT‐PEDOT:PSS contributed significantly to improving device performance, and this method was universal and applicable to various color emitting Pero‐LEDs, including pure blue (472 nm), cyan (500 nm), and green (515 and 525 nm). For all of these color emitting Pero‐LEDs based on UT‐PEDOT:PSS, around twofold enhancements of luminance were achieved, and according to the statistical results, the EQEs of Pero‐LEDs based on 3D, quasi‐3D, and quasi‐2D perovskites obtained enhancements of 42%, 87%, and 111%, respectively. The maximum EQE reached 17.6%, 15.0%, and 6.8% for Pero‐LEDs based on 3D, quasi‐3D, and quasi‐2D, respectively.

As shown in **Figure** **1**a, we prepared three sets of Pero‐LEDs with the different interfaces of perovskite and substrates, that is, perovskite/PEDOT:PSS, perovskite/ITO, and perovskite/UT‐PEDOT:PSS. The conventional thick PEDOT:PSS is supposed to block electrons effectively but will induce some loss of photons, that is, low LEE, mostly due to its absorbance in the visible light region. On the one hand, we can increase the LEE by removing the PEDOT:PSS layer; however, this in returns cause severe non‐radiative recombination in the interface of perovskite/ITO. We propose to realize the balance of increasing hole injection capability and the loss of photons by simply reducing the thickness of PEDOT:PSS, that is, preparing an ultrathin layer of PEDOT:PSS. Here, we prepared the conventional thick PEDOT:PSS using the well‐documented method, and we developed a water stripping method to prepare the UT‐PEDOT:PSS. The thickness of PEDOT:PSS (Figure 1b) and UT‐PEDOT:PSS (Figure 1c) were measured using the film thickness gauges (ThetaMetrisis FR‐pRo VIS/NIR) (Figure S1, Supporting Information). The thickness of PEDOT:PSS declined dramatically from 54.7 to 6.9 nm after water stripping treatment. Atomic force microscope (AFM), X‐ray photoelectron spectroscopy (XPS) and ultraviolet photoelectron spectrometer (UPS) measurements were carried out to investigate the surface morphology, composition, and energy level of the as‐prepared PEDOT:PSS films, as these properties will have great influence in device performance. As indicated in the AFM images (Figure S2, Supporting Information), the ITO sample shows a smooth surface with packed particles, whereas PEDOT:PSS and UT‐PEDOT:PSS samples show uniform and smoother films coating on ITO, but the sample of UT‐PEDOT:PSS reveals an ultra‐thin thickness as it almost reveals the morphology of the bottom layer of ITO. And according to the current density–voltage (*J–V*) curves (Figure S3, Supporting Information) of Pero‐LEDs based on 3D perovskite, little leakage current was observed at low bias when the thickness of PEDOT:PSS was declined by the water stripping method. These results indicate that the UT‐PEDOT:PSS could fulfill the targets of surface smoothing and blocking leakage current. The XPS (Figure 1d) shows the S(2p) peaks spectra of PEDOT:PSS and UT‐PEDOT:PSS. There are two signal bands; the lower band between 166 and 171 eV is derived from the signal of PSS, and the higher band between 160 and 166 eV is attributed to the signal from PEDOT.^[^ ^29^ ^]^ Since the relative peak intensities can be used to determine the ratio of PSS and PEDOT, the UT‐PEDOT:PSS shows a remarkable reduction of the PSS‐to‐PEDOT ratio. The PSS‐to‐PEDOT ratio decreased from 0.66 to 0.54 after water stripping treatment. Taking the insulation characteristic of PSS into consideration, the reduction of PSS‐to‐PEDOT ratios should contribute to enhance the electrical conductivity of the ultrathin PEDOT:PSS. The UPS data (Figure 1e) show a deeper HOMO level (from −5.1 to −5.2 eV) after water stripping treatment, which is more matched to the VB_max_ of perovskite and should induce a better hole injection process. To compare the charge injection efficiency between the interface of HTL/perovskite and ETL/perovskite, hole‐only devices (ITO/PEDOT:PSS(54.7/6.9 nm)/perovskite/MoO_3_/Au), and electron‐only devices (ITO/B3PYMPM/perovskite/B3PYMPM/LiF/Al) were fabricated and the corresponding current density–voltage (*J*–*V*) curves were recorded. Figure 1f shows a higher current density for electron‐only devices than that of the hole‐only devices based on original PEDOT:PSS, indicating an imbalance of charge injection. However, the current density of the hole‐only devices based on UT‐PEDOT:PSS increased remarkably, and therefore a well‐balanced charge injection was obtained finally. Time‐resolved photoluminescence (TRPL) decay curves (Figure 1g) display a longer lifetime for perovskite coated on UT‐PEDOT:PSS (τ = 6.3 ns) than that on original PEDOT:PSS (τ = 1.3 ns). This result may be attributed to the chemical environment change caused by the reduction of insulating PSS, which leads to less non‐radiative recombination at the perovskite/HTL interface.

**Figure 1:**
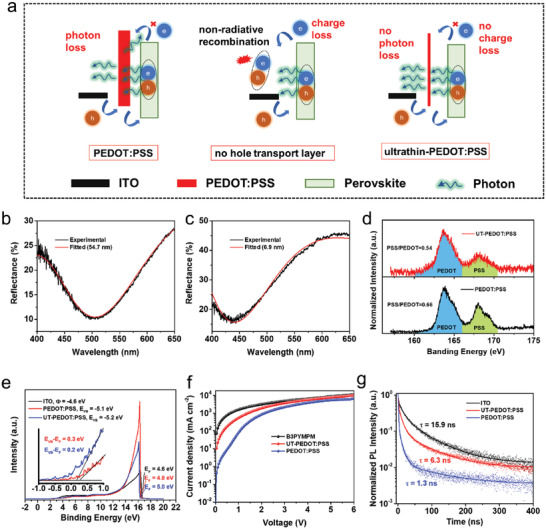
a) Schematic illustration of the charge injection and recombination process, and transportation routes of photons in the Pero‐LEDs with different perovskite/substrate interfaces, that is, perovskite/PEDOT:PSS, perovskite/ITO, and perovskite/UT‐PEDOT:PSS. Thickness measurement of b) PEDOT:PSS and c) UT‐PEDOT:PSS using FR‐pRo VIS/NIR. d) X‐ray photoelectron spectra (S(2p) peaks) of PEDOT:PSS and UT‐PEDOT:PSS. e) UPS spectra of ITO, PEDOT:PSS, and UT‐PEDOT:PSS. f) *J–V* curves of the electron‐only device based on B3PYMPM, and hole‐only devices based on original PEDOT:PSS and UT‐PEDOT:PSS. g) Time‐resolved photoluminescence decay curves of perovskite films on different substrates.

To evaluate the influence of the thickness of the PEDOT:PSS layer on the LEE of the Pero‐LEDs, optical simulations were conducted with the Chance–Prock–Silbey (CPS) model by the integrated commercial software of Setfos (4.6.10 from Fluxim AG). The model constructed in Setfos includes Glass (1.1 mm)/ITO (150 nm)/PEDOT:PSS (0–60 nm)/perovskite (80 nm)/B3PYMPM (50 nm)/LiF (1 nm)/Al (60 nm) (Figure S4a, Supporting Information), and the thickness of each layer of the device was obtained from the cross‐sectional scanning electron microscope (SEM) image (Figure S4b, Supporting Information). And the optical constants of various materials were measured and are provided in Figure S5, Supporting Information. The quasi‐3D Pero‐LEDs were modeled whose emitting wavelength was 515 nm and the general design principles and analysis can be applied to all three sets of the Pero‐LEDs. The corresponding optical constants and the electroluminescence (EL) spectrum (**Figure** **2**a) of perovskite were integrated into the model. The optical constants (the real and imaginary part of the refractive index) were extracted from the best fitting model by an ellipsometer (Horiba UVISEL), in the range of wavelength from 350 to 900 nm. These parameters were then input into the Setfos consistent with the experiments. Unless otherwise stated, the dipole source was placed in the center of the emitting layer and the dipole orientation was selected as isotropical to simulate the real device. The glass substrate was set to be incoherent due to its large thickness for the actual device. To clarify the underlying mechanism of the thickness variation of the PEDOT:PSS induced LEE change, power dissipation channels of the Pero‐LEDs were examined including the outcoupled mode, substrate/guided mode, absorption loss, and evanescent coupling. These modes were originally defined in the OLEDs by their in‐plane wave vectors to quantitatively describe each power dissipation channel.^[^ ^30^, ^31^ ^]^ In our case, the outcoupled mode has the same definition as the LEE and is used to evaluate the portion of escaped photons outside the Pero‐LED to the total emitted photons in the active layer. We assumed the IQE to be 100% in the simulation. The substrate or guided mode include the internally created photons that experience total internal reflection (TIR) at the substrate/air and other inner interfaces within the Pero‐LEDs and are trapped inside the glass and other functional layers. The absorption loss reflects the radiated power from the active layer being absorbed in the Pero‐LED. The evanescent coupling measures the radiated power evanescently coupling to surface plasmons and the longitudinal oscillation of electrons at the metal/dielectric interfaces.

**Figure 2:**
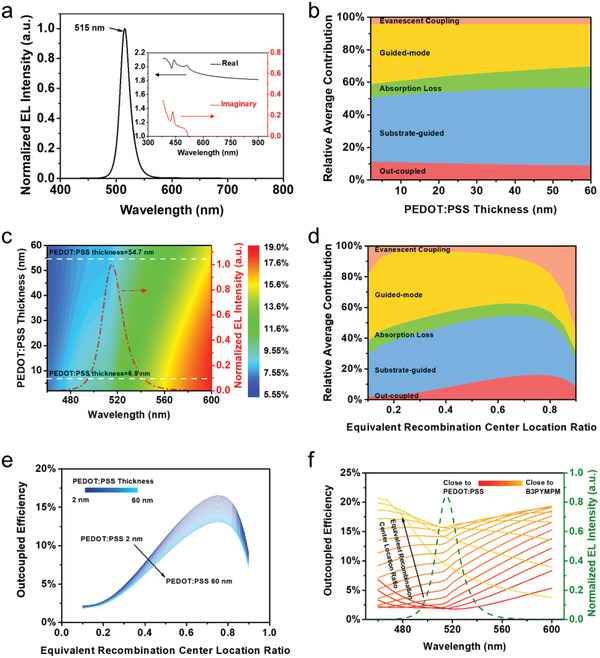
Theoretical analysis and modeling of the influence of the thickness of the PEDOT:PSS on the LEE of the Pero‐LEDs. a) Electroluminescence spectra with inserted refractive index of quasi‐3D perovskite. b) Power dissipation channels of the Pero‐LEDs at different PEDOT:PSS thicknesses. c) The LEE as a function of the PEDOT:PSS thickness and emission wavelength. d) The power dissipation channels variation as the equivalent recombination center location change at a fix PEDOT:PSS thickness of 6.9 nm. e) LEE changes with equivalent recombination center location for various PEDOT:PSS thickness and f) the LEE spectra for various equivalent recombination center locations with the electroluminescence spectra overlaid.

The LEE is highly dependent on thickness of the PEDOT:PSS in Pero‐LEDs. As shown in Figure 2b, in general, the increase of the PEDOT:PSS thickness leads to a reduction of the portion of the outcoupled mode. This is mainly because the extinction coefficients of the PEDOT:PSS are nonzero (Figure S5, Supporting Information) within the green light region. As the equivalent recombination center location remains unchanged at the center of the active layer, the evanescent coupling maintains almost the same value. Besides, since the refractive indexes of the functional layers of the Pero‐LED will not change as the increment of the thickness of the PEDOT:PSS, the critical angles at the inner interfaces defined by TIR of the light escaping cone of the emitted light remain undisturbed. Therefore, the sum of the guide and substrate modes remain almost the same values as the increase of the thickness of the PEDOT:PSS. The LEE as a function of the incident wavelength and the thickness of the PEDOT:PSS is also provided in Figure 2c with the EL spectrum overlaid. As the thickness of the of the PEDOT:PSS decreases, the device structure becomes more favorable for the photons to escape outside within the wavelengths covered by the EL spectrum, and therefore leads to higher LEE corresponding to the experimental measurement of higher EQE.

The influence of the equivalent recombination center location on the LEE was also evaluated, and the results are shown in Figure 2d–f. The equivalent recombination center location is defined by the ratio of the position of the equivalent recombination center to the overall thickness of the quasi‐3D perovskite. It ranges from 0 to 1 indicating the movement of the position of the equivalent recombination center from top (close to PEDOT:PSS) to the bottom (close to the B3PYMPM) within the emitting layer. Figure 2d shows when the thickness of the PEDOT:PSS is 6.9 nm, the LEE is greatly improved from 10.9% to a maximum value of 16.1% with the change of the equivalent recombination center location from 0.5 to 0.75. A similar trend is found for various thicknesses of PEDOT:PSS as shown in Figure 2e. Another noticeable feature is the significant change of the evanescent coupling when the equivalent recombination center locates at either top or the bottom interfaces of the quasi‐3D perovskite. This is attributed to the strong excitation of the electromagnetic modes propagating along the interfaces of the emitting layer and the transport layers.^[^ ^32^, ^33^ ^]^ The outcoupled efficiency spectra were also evaluated as a function of the equivalent recombination center location as shown in Figure 2f. It is found that when the equivalent recombination center is close to either top or bottom sides of the emitting layer the outcoupling efficiencies obviously decrease across the emission spectrum. On the contrary, the optimal recombination center location will lead to a local maximum value for the outcoupling efficiency where the photons having the wavelengths within the quasi‐3D perovskite emission spectrum.

Before the real Pero‐LEDs fabrication, the characteristics of 3D, quasi‐3D, and quasi‐2D perovskite films spin‐coated on different substrates were studied first. The SEM images in Figure S6, Supporting Information, show the smooth and dense perovskite films coated on different substrates. Specifically, the 3D perovskite coated on UT‐PEDOT:PSS and PEDOT:PSS show remarkable smaller crystals and negligible pinholes compared to that coated on ITO. These findings indicate that water stripping treatment had little side impact on perovskite crystallization, and we can still get high quality perovskite coating on UT‐PEDOT:PSS. According to the X‐ray diffraction spectra in Figure S7, Supporting Information, the identical peaks of 3D perovskite on PEDOT:PSS and UT‐PEDOT:PSS verified that there was no negative impact for perovskite crystallization after water stripping treatment. The pure phase (CsPbBr_3_) of 3D perovskite coated on UT‐PEDOT:PSS and PEDOT:PSS indicated better perovskite films were obtained, whereas mixed phases (CsPbBr_3_ and Cs_4_PbBr_6_) were observed in the sample coated on ITO. The photoluminescence and absorption spectra in Figure S8, Supporting Information, show a sequential blue shift of emission peaks and absorption of 3D, quasi‐3D, and quasi‐2D perovskite. This indicates that the energy bandgaps of perovskites are well adjusted and Pero‐LEDs with different color emissions can be fabricated using the as‐prepared perovskite films. And the PLQY of different perovskites were measured using the integrating sphere method, and the PLQY of 3D, quasi‐3D, and quasi‐2D perovskites are 75%, 71%, and 60%, respectively. All of the devices were fabricated with a general Pero‐LED structure (ITO/HTL(PEDOT:PSS/UT‐PEDOT:PSS)/perovskite/B3PYMPM/LiF/Al), and the cross‐sectional SEM (**Figure** **3**a) shows the well‐defined layer‐by‐layer structure. The band alignment of all functional layers is shown in Figure 3b, the HOMO level of UT‐PEDOT:PSS is slightly lower than that of PEDOT:PSS, which should contribute to a better hole injection process. Figure 3c–e shows the EL spectra and emitting device photographs of the 3D, quasi‐3D, and quasi‐2D based Pero‐LEDs, respectively. The ultra‐stable EL peaks are located at 525, 515, and 500 nm, and the full width at half maximum are about 20, 20, and 24 nm, respectively. To fully investigate the performance improvement effect by UT‐PEDOT:PSS, we fabricated several batches of Pero‐LEDs based on 3D, quasi‐3D, and quasi‐2D perovskites. The corresponding histograms of peak luminance spectra (Figure 3f–h) show a dramatic enhancement of luminance for UT‐PEDOT:PSS devices. Specifically, according the statistical results, there are about 84%, 69%, and 115% of enhancement for 3D, quasi‐3D, and quasi‐2D Pero‐LEDs, respectively. This outstanding increment of luminance should be attributed to a higher LEE and a better hole injection of interface of UT‐PEDOT:PSS and perovskite, which are consistent with the previous experimental and simulation results. The histograms of peak EQE spectra (Figure 3i–k) also show a remarkable enhancement of EQE for UT‐PEDOT:PSS devices, and there are about 42%, 87%, and 111% of enhancement for 3D, quasi‐3D, and quasi‐2D Pero‐LEDs, respectively. As shown in Figure S9, Supporting Information, the maximum EQEs of 3D, quasi‐3D, and quasi‐2D Pero‐LEDs are 17.6%, 15.0%, and 6.8%, respectively. To further verify the capability of UT‐PEDOT:PSS to enhance the performance of colorful Pero‐LEDs, the pure blue Pero‐LEDs were also fabricated, and the EL peaks (emission at 472 nm) were recorded in Figure S10a, Supporting Information. The luminance–voltage curves in Figure S10b, Supporting Information, demonstrated a lower turn‐on voltage (2.8 and 3.4 eV for Pero‐LEDs based on UT‐PEDOT:PSS and PEDOT:PSS, respectively) and a more than twofold enhancement of luminance for Pero‐LEDs based on UT‐PEDOT:PSS. And the operation lifetimes of Pero‐LEDs were also measured at a constant current density of 200 mA cm^−2^. The results in Figure S11, Supporting Information, demonstrate that the lifetime of quasi‐3D Pero‐LEDs based on original PEDOT:PSS was *T*_50_ = 54.5 min, and the device based on UT‐PEDOT:PSS was increased to *T*_50_ = 67.8 min.

**Figure 3:**
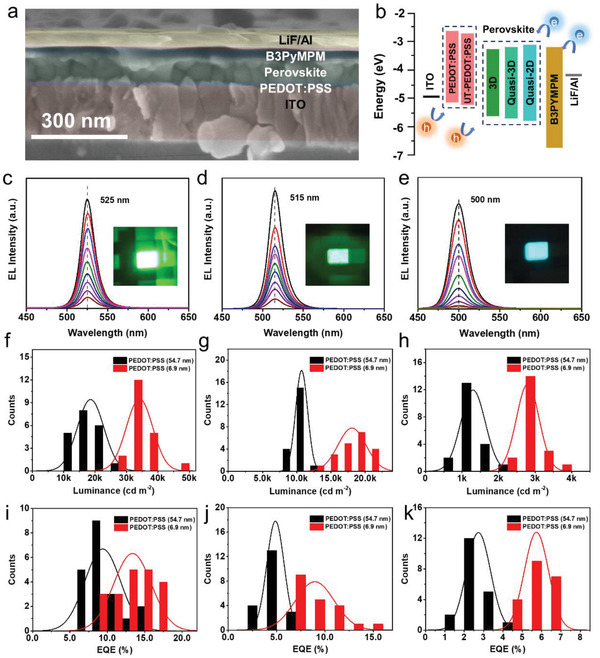
Fabrication and performance evaluation of Pero‐LEDs with different perovskite/substrate interfaces. a) Cross‐sectional SEM image of one Pero‐LEDs device. b) Band alignment diagram of Pero‐LEDs with various perovskite emitting layers, that is, 3D, quasi‐3D, and quasi‐2D. c–e) Electroluminescence spectra with inserted photographs of emitting devices, f–h) statistic histograms of peak luminance, and i–k) statistic histograms of peak EQE of Pero‐LEDs with 3D perovskite, quasi‐3D perovskite, and quasi‐2D perovskite emitting layers.

In summary, we have demonstrated a facile method to prepare UT‐PEDOT:PSS and proved that both the electrical and optical properties for fabricating Pero‐LEDs have been significantly improved using experimental and simulation results. Moreover, the UT‐PEDOT:PSS was used to fabricate and enhance performance of colorful emitting Pero‐LEDs (green (525 and 515 nm), cyan (500 nm), and pure blue (472 nm)). Due to the conductivity enhancement and decline of HOMO energy level, there was a more balanced charge injection for Pero‐LEDs based on UT‐PEDOT:PSS. Together with the increased LEE, both of the luminance and EQE were enhanced dramatically. As a result, the EQE_max_ of Pero‐LEDs based on 3D, quasi‐3D, and quasi‐2D perovskite were elevated to 17.6%, 15%, and 6.8%, respectively. Our work provides a universal method to fabricate various color‐emitting and high‐efficiency Pero‐LEDs, which could make a contribution to bridging the gap between Pero‐LEDs and other mature technologies.

## Conflict of Interest

The authors declare no conflict of interest.

## Supporting information

Supporting Information
