# Supplementary material for: Ultrathin PEDOT:PSS Enables Colorful and Efficient Perovskite Light‐Emitting Diodes
Source: Adv Sci (Weinh). 2020 Apr 13;7(11):2000689. doi: 10.1002/advs.202000689 (PMC7284212; doi:10.1002/advs.202000689)
Supplement: Supplementary file 1 — Supporting Information [file ADVS-7-2000689-s001.pdf]

Copyright WILEY-VCH Verlag GmbH & Co. KGaA, 69469 Weinheim, Germany, 2020.

ADVANCED SCIENCE Open Access

Supporting Information

for *Adv. Sci.,* DOI: 10.1002/advs.202000689

Ultrathin PEDOT:PSS Enables Colorful and Efficient Perovskite Light-Emitting Diodes

Jianxun Lu, Wenjing Feng, Guanding Mei, Jiayun Sun, Chuanzhong Yan, Di Zhang, Kebin Lin, Dan Wu, Kai Wang,* and Zhanhua Wei**

Copyright WILEY-VCH Verlag GmbH & Co. KGaA, 69469 Weinheim, Germany, 2020.

Supporting Information

Ultrathin PEDOT:PSS Enables Colorful and Efficient Perovskite Light-emitting Diodes

Jianxun Lu[1], Wenjing Feng[1], Guanding Mei[3], Jiayun Sun[3], Chuanzhong Yan[1], Di Zhang[1], Kebin Lin[1], Dan Wu[2,3,], Kai Wang[3,]*, and Zhanhua Wei[1,]**

Mr. J. Lu, Miss W. Feng, Mr. C. Yan, Miss D. Zhang, Mr. K. Lin, Prof. Z. Wei
Institute of Luminescent Materials and Information Display, College of Materials Science & Engineering, Huaqiao University, Xiamen 361021, P.R. China
E-mail: weizhanhua@hqu.edu.cn
Prof. D. Wu
Academy for Advanced Interdisciplinary Studies, South University of Science and Technology of China Shenzhen 518055, P.R. China
E-mail: wud@sustech.edu.cn
G. Mei, J. Sun, Prof. D. Wu, Prof. K. Wang
Department of Electrical & Electronic Engineering, South University of Science and Technology of China Shenzhen 518055, P.R. China
E-mail: wangk@sustc.edu.cn

Experimental Section

Materials. Unless otherwise stated, all chemicals were purchased from Sigma-Aldrich and used as received.

Preparation of perovskite precursor. $CsPbBr_3$ was prepared firstly by dissolving 10 mmol of $PbBr_2$ (≥98 %) in 8 mL of hydrobromic acid (48 wt.% in H_2O, ≥99.99 %), then 10 mmol of CsBr (dissolved in 3 mL of H_2O) was added drop by drop, producing an orange precipitate. The precipitate was filtered, washed twice using ethanol, and dried at 60 ℃ in a vacuum oven for 12 h before use. 2-phenylethanolamine bromide (PEABr) was prepared by reacting 6.3

mL of phenethylamine (≥99 %) and 8.5 mL of hydrobromic acid (48 wt.% in H_2O, ≥99.99 %) in an ice bath for 30 min with vigorous stirring. The solvent was removed using rotary evaporation at 50 ℃ to obtain a white platy crystals. For purification, the as-prepared PEABr was re-dissolved in ethanol and precipitated with diethyl ether, and this prosses was repeated once again. Finally, the white powder was collected by filtration and dried at 60 ℃ in a vacuum oven for 12 h before use.

$CsPbBr_3$ and methylamine (MABr) with molar ratio 1:1 were dissolved with DMSO (anhydrous, ≥99.9 %) to form the 3D Perovskite precursor solution. The concentration for $CsPbBr_3$ was 0.5 M. The solutions should be stirred continuously at room temperature for at least 12 h before use. Quasi-3D and quasi-2D perovskite precursor solutions were prepared by dissolving $CsPbBr_3$ and PEABr with molar ratio 1:0.6 and 1:1.0 respectively in DMSO under continuous stirring for at least 2 h at room temperature, keeping the molar concentration of Pb^{2+} at 0.3 M. And the pure blue perovskite was prepared by dissolving $CsPbBr_3$ and 2-phenylethanolamine chloride (PEACl) with molar ratio 1:1 in DMSO under vigorous stirring for at least 2 h at room temperature, keeping the molar concentration of Pb^{2+} at 0.2 M.

Fabrication of perovskite LEDs. The patterned ITO-coated glasses were ultrasonically cleaned in detergent solution, deionized water, acetone, isopropyl alcohol, and ethanol, and then dried with compressed N_2. The substrates were further cleaned with UV-Ozone cleaner (Novascan, PSD) for 30 min before use. A hole transporter layer (HTL) was prepared by spin-coating using PEDOT:PSS (Clevios PVP AI4083) at 4,000 r.p.m. for 40 s, and baked at

130 ℃ for 15 min in ambient air. To prepare the ultrathin PEDOT:PSS, 200 μL of deionized water was spin-coated on the as-prepared PEDOT:PSS film drop by drop at 5,000 r.p.m. for 30 s, and the substrate was baked again at 130 ℃ for 15 min in ambient air. After cooling to room temperature, the substrates were transferred into a N_2-filled glovebox. For the emitting material layers, 30 μL of perovskite precursor solution was spin-coated onto the HTL at 2,000 r.p.m. for 60 s, antisolvent (500 μL of toluene for 3D perovskite, 50 μL of Acetone for quasi-3D, quasi-2D, and pure blue perovskite) was added rapidly at 30 s before spin-coating ending. Finally, a 50-nm-thick layer of 4,6-Bis(3,5-di-3-pyridinylphenyl)-2-methylpyrimidine (B3PYMPM, Lumtec, Taiwan), a 1-nm-thick layer of LiF and an 60-nm-thick layer of Al were deposited using a thermal evaporation system under a vacuum of $<1.0\ x10^{-4}$ Pa. The active area was 3 mm^2 (2 mm × 1.5 mm), which was defined by the overlapping area of ITO and Al electrodes.

Pero-LEDs characterizations. All of the devices were measured in a N_2-filled glovebox with a home-made test socket. A Keithley 2400 instrument was used to measuring Current density (J)-Voltage (V) data from 0 to 6 V with a step voltage of 0.2 V and a delay time of 1 s. Simultaneously, the luminance was measured with a luminance meter (Konica Minolta, CS-200), and electroluminescence (EL) spectra were recorded by a Flame spectrometer (Ocean Optic). The current efficiency was calculated by dividing the luminance by current density, and the EQE was calculated using Lambertian emission profiles and obtained the EL spectrum.

Perovskite film characterizations. The perovskite film morphology was characterized by SEM (Hitachi S-8000 scanning electron microscope) and AFM (Bruker Multimode 8). XRD patterns were obtained from a D8 Advance diffractometer (Bruker AXS). UV-vis absorption and steady-state photoluminescence spectra were obtained using a Flame spectrometer (Ocean Optics) in a N_2-filled glovebox. The XPS and UPS spectra were obtained on an expanding X-ray Photoelectron Spectrometer (Thermofisher ESCALAB Xi+). Time-resolved PL decay curves were measured by a Fluorescence spectrophotometer (Edinburgh FLS920) with a pulsed excitation laser of 405 nm.

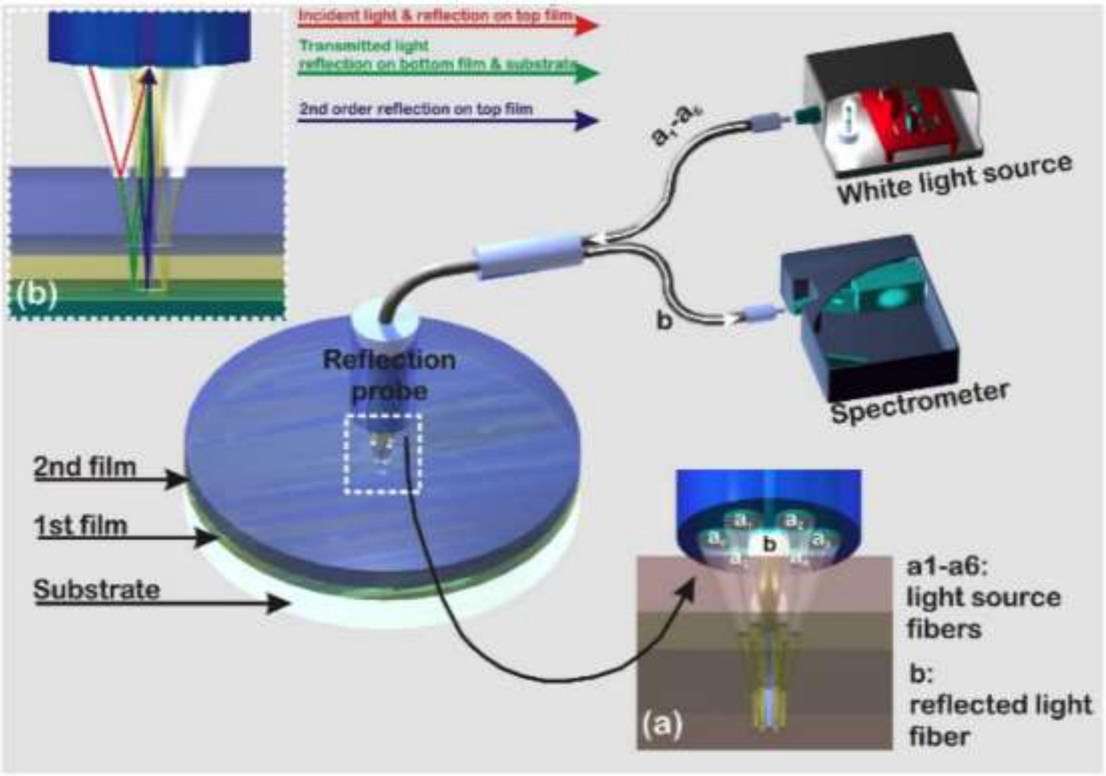

Figure S1. Schematic of Film Thickness Gauges (ThetaMetrisis FR-pRo VIS/NIR).

Film Thickness Gauges are used to measure the thickness and optical constants (n&k) of dielectric, resists, metal and semiconductor thin films through white light reflectance spectroscopy (WLRS), an accurate and non-destructive methodology.

WLRS methodology involves a light source, a PC-driven miniaturized spectrometer, and a reflection probe. The light emitted from the light source is guided onto the sample under investigation through a reflection optical probe fibers a1-a6, (Figure S1a, Supporting Information). The beam from the light source interacts with the sample (Figure S1b, Supporting Information) and produces a reflectance signal. The reflection optical probe collects the reflected beam (through the fiber b), directing it to the spectrometer. Due to the different optical paths the various beams have undergone, interference fringes are monitored

on the spectrometer. By fitting the reflectance spectrum with appropriate physical models and algorithms the film thicknesses and the refractive indices of the films could be calculated.

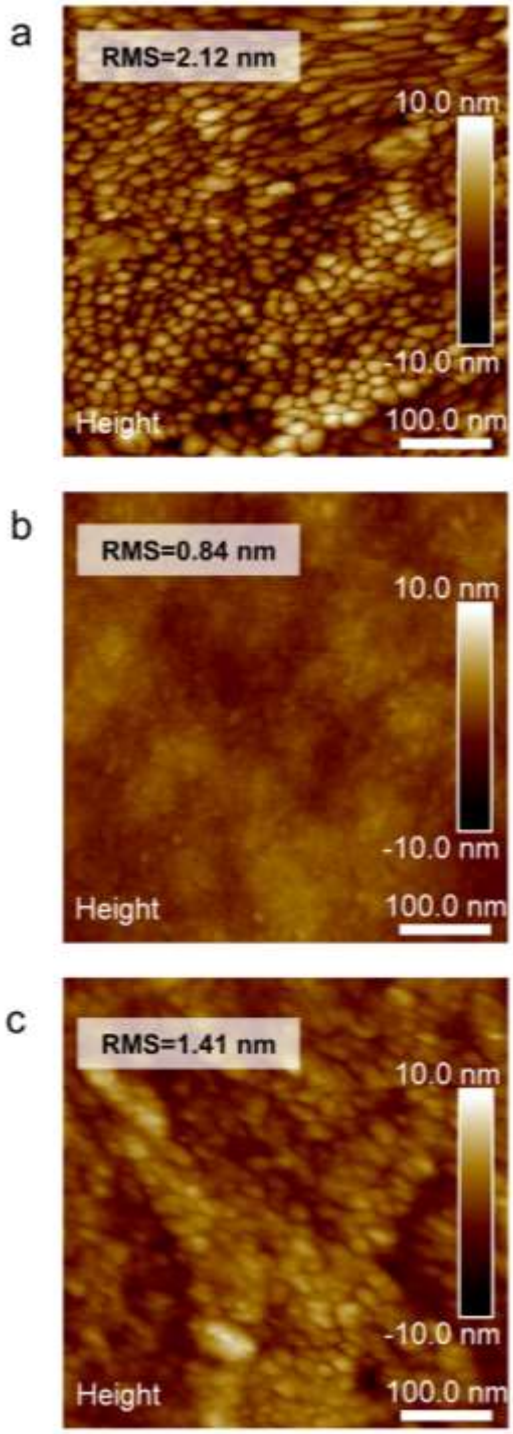

Figure S2. Atomic force microscope (AFM) of a) ITO, b) PEDOT:PSS, c) ultrathin PEDOT:PSS.

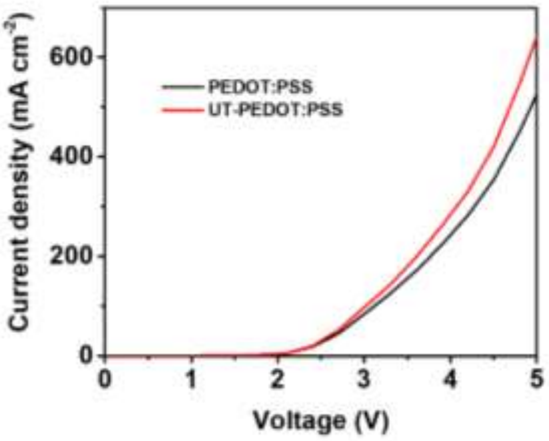

Figure S3. The Current density-Voltage (*J*-*V*) curves of 3D Pero-LEDs based on PEDOT:PSS and UT-PEDOT:PSS.

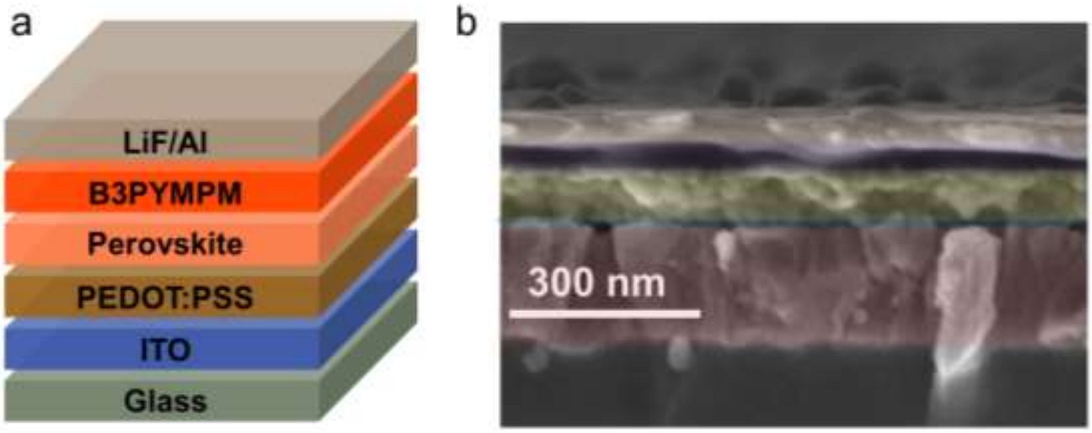

Figure S4. Illustration of actual Pero-LED structure a), and the corresponding cross-sectional SEM image b) of device based on quasi-3D Pero-LED.

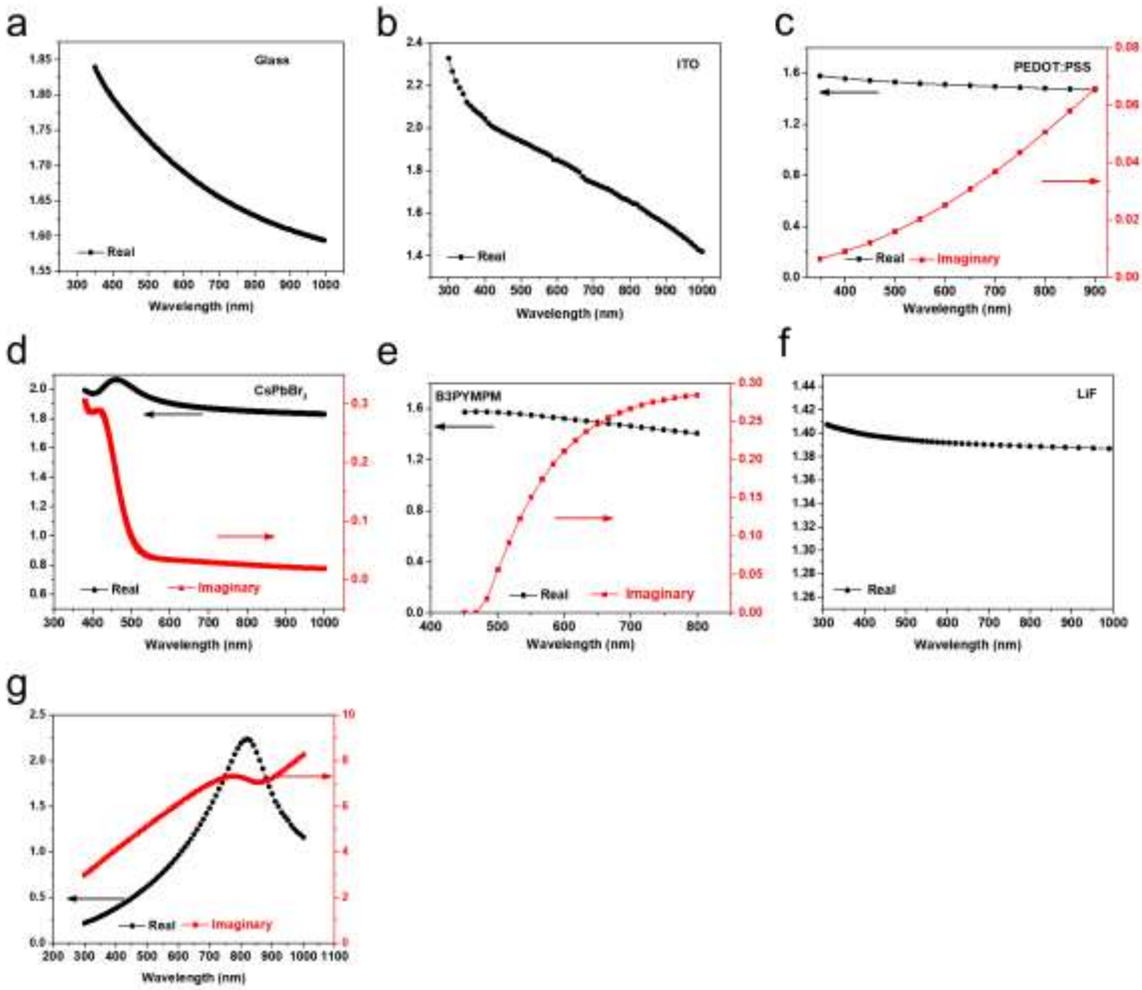

Figure S5. Refractive index of a) Glass, b) ITO, c) PEDOT:PSS, d) CsPbBr3, e) B3PYMPM, f) LiF and g) Al.

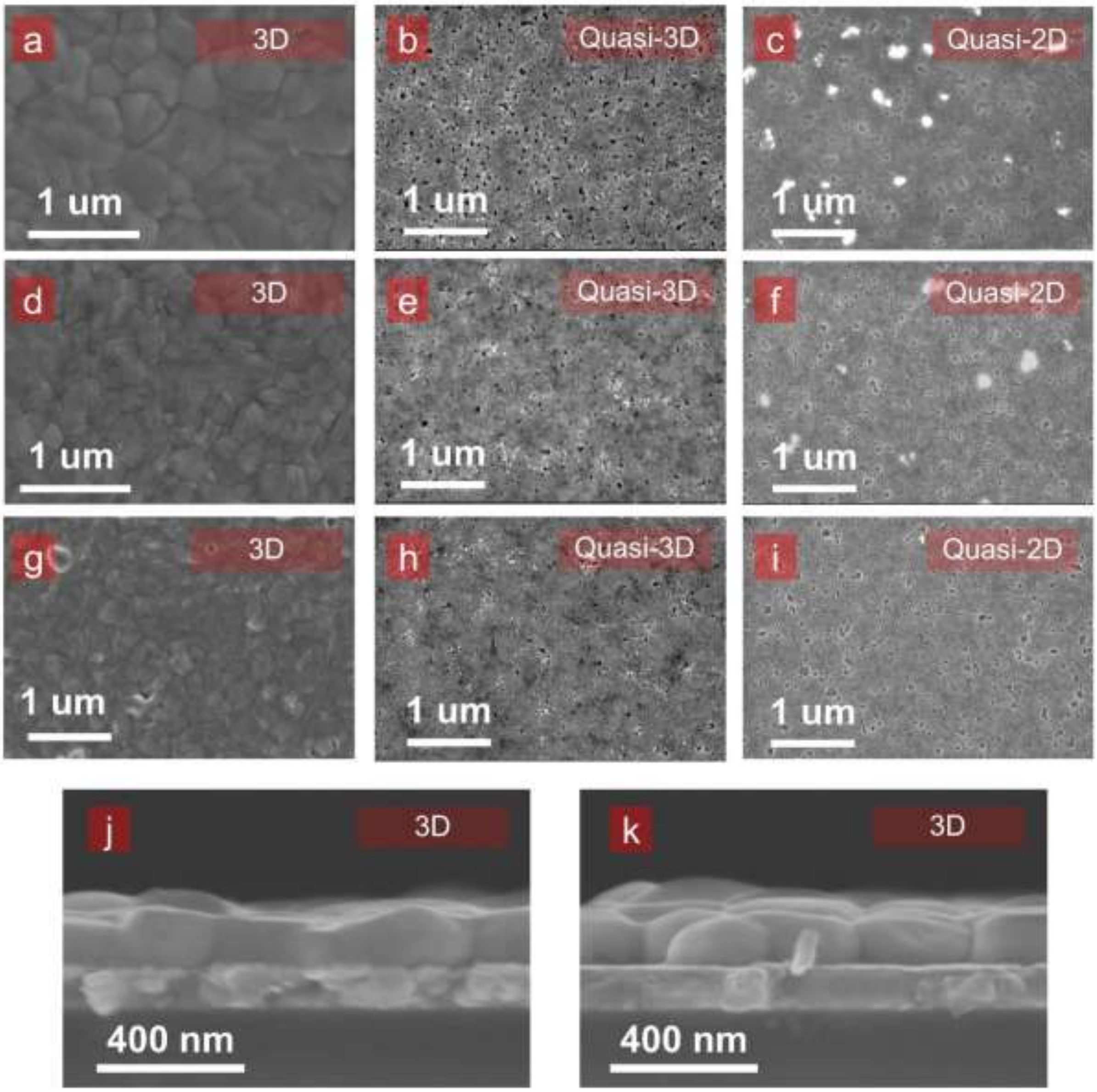

Figure S6. Scanning electron microscope (SEM) images of 3D, quasi-3D and quasi-2D perovskites prepared on ITO (a-c), ultrathin PEDOT:PSS (d-f), and PEDOT:PSS (g-i) respectively. Cross-sectional SEM images (i,k) of 3D perovskite spin-coating on ultrathin PEDOT:PSS.

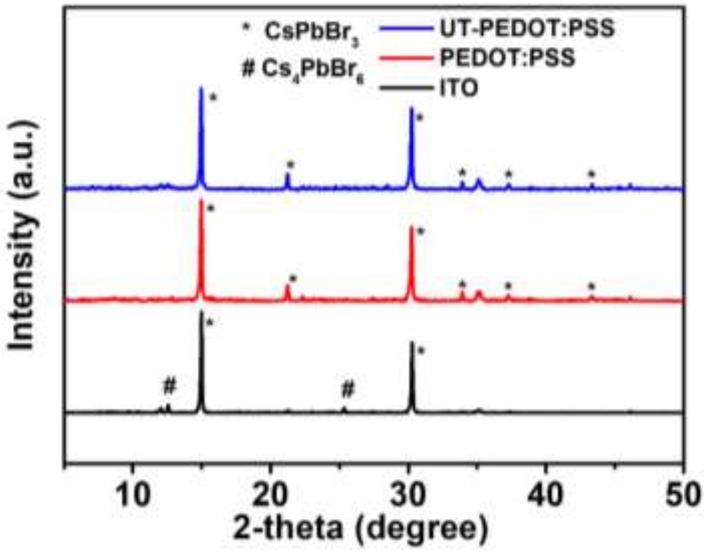

Figure S7. XRD spectra of 3D perovskite spin-coated on ITO, PEDOT:PSS, and ultrathin PEDOT:PSS.

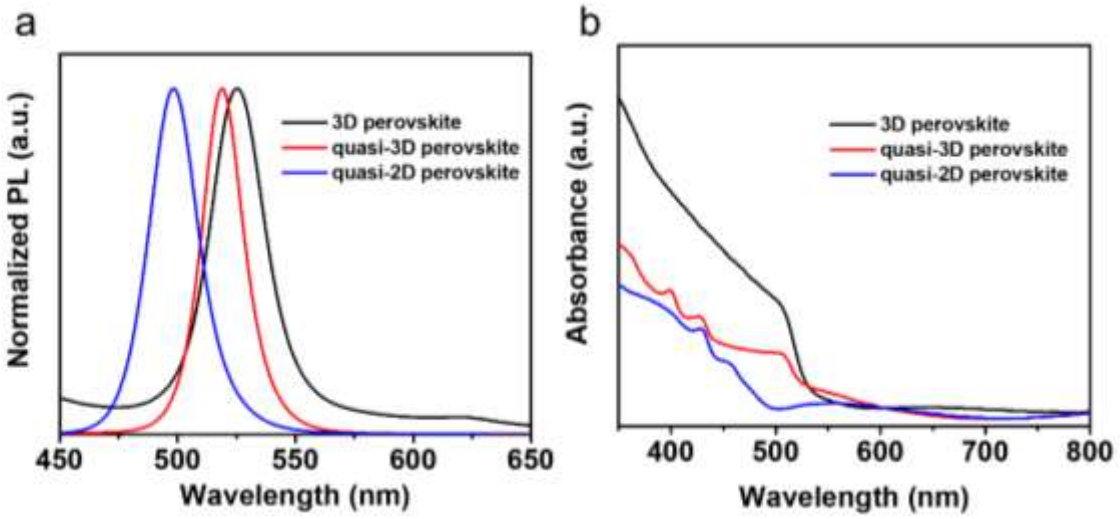

Figure S8. a) Normalized PL and b) absorption spectra of 3D perovskite, quasi-3D perovskite, and quasi-2D perovskite spin-coated on ultrathin PEDOT:PSS.

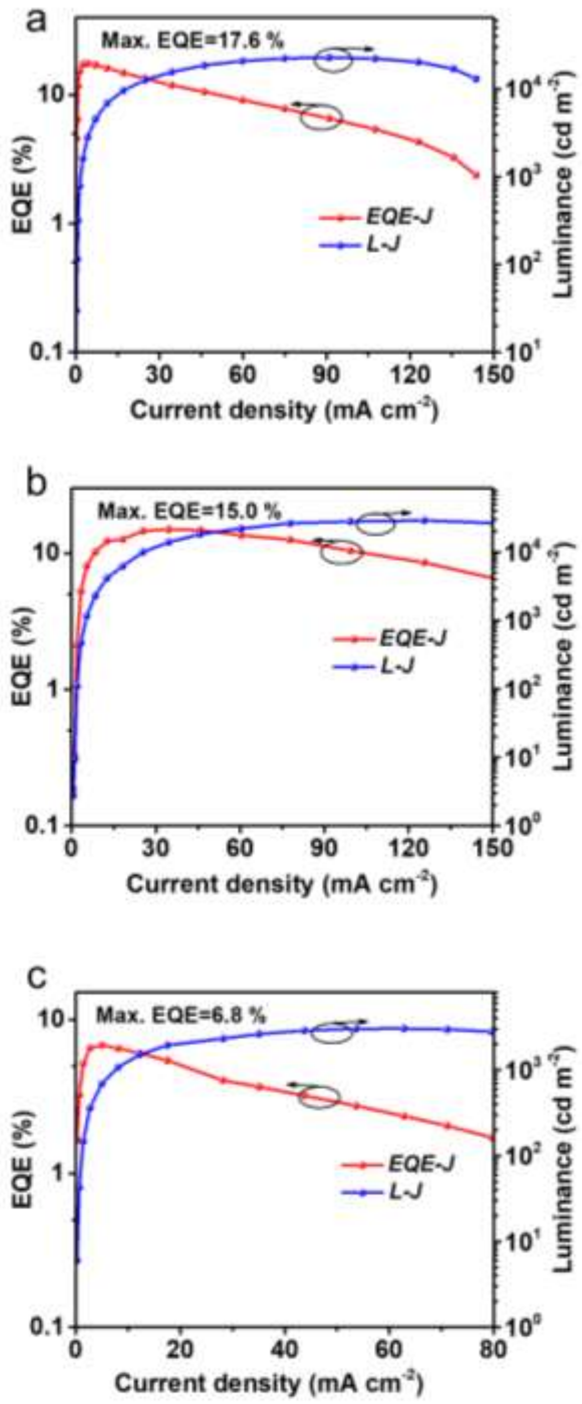

Figure S9. *EQE*-Current density (*J*)-Luminance (*L*) curves of a) 3D Pero-LED, b) quasi-3D Pero-LED and c) quasi-2D Pero-LED.

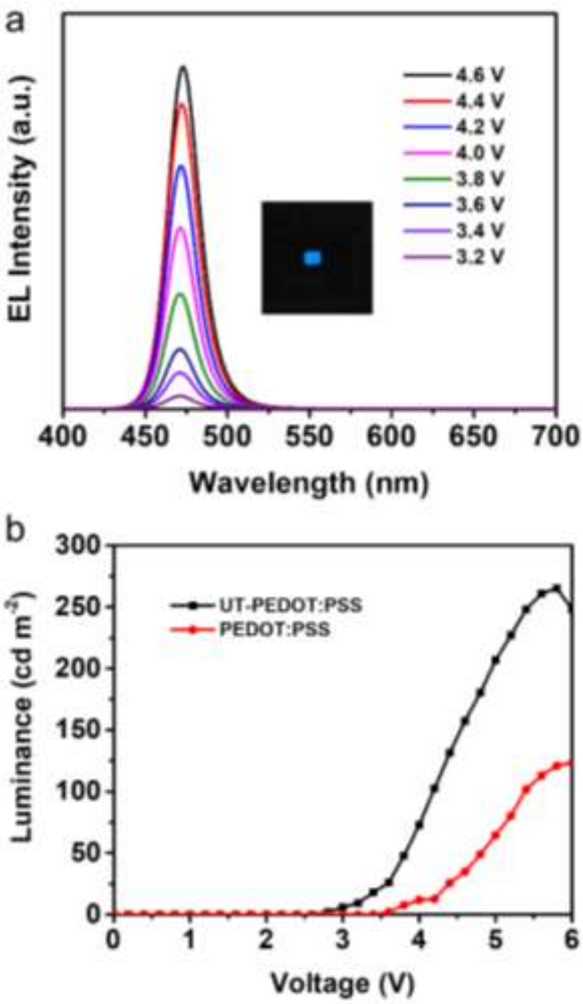

Figure S10. The a) EL spectra with inserted photograph and b) Luminance-Voltage curves of pure blue Pero-LEDs.

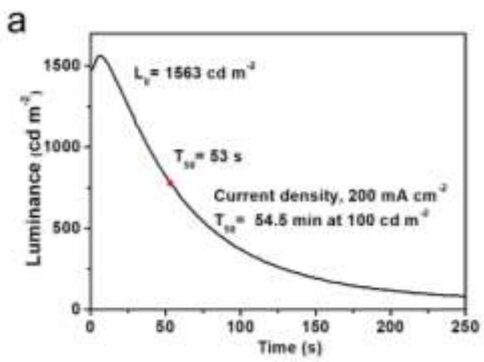

Figure S11. The operation lifetime of quasi-3D Pero-LEDs based on a) PEDOT:PSS and b) UT-PEDOT:PSS.
